# Antiviral activity of ginseng extract against respiratory syncytial virus infection

**DOI:** 10.3892/ijmm.2014.1750

**Published:** 2014-04-22

**Authors:** JONG SEOK LEE, EUN-JU KO, HYE SUK HWANG, YU-NA LEE, YOUNG-MAN KWON, MIN-CHUL KIM, SANG-MOO KANG

**Affiliations:** 1 Center for Inflammation, Immunity and Infection, Institute for Biomedical Sciences, Georgia State University, Atlanta, GA 30303, USA; 2 Animal and Plant Quarantine Agency, Anyang, Gyeonggi-do, Republic of Korea

**Keywords:** *Panax ginseng*, respiratory syncytial virus, viral replication, interferon

## Abstract

*Panax ginseng* has been known to have a number of immuno-modulatory effects. In this study, we investigated whether *Panax* Korean red ginseng extract (KRGE) has *in vitro* and *in vivo* antiviral effects on respiratory syncytial virus (RSV) infection. KRGE improved the survival of human lung epithelial cells against RSV infection and inhibited RSV replication. In addition, KRGE treatment suppressed the expression of RSV-induced inflammatory cytokine genes (IL-6 and IL-8) and the formation of reactive oxygen species in epithelial cell cultures. Oral administration of mice with KRGE resulted in lowering lung viral loads after RSV infection. Additionally, the *in vivo* effects of KRGE showed an enhanced level of interferon-γ (IFN-γ) producing dendritic cells subsequent to RSV infection. Taken together, these results suggested that KRGE has antiviral activity against RSV infection.

## Introduction

Respiratory syncytial virus (RSV) is a non-segmented, negative-stranded RNA virus and a member of the *Paramyxoviridae* family. RSV is the leading cause of serious respiratory infections in children as well as in elderly and immune-suppressed individuals (1,2). The role of reactive oxygen species (ROS) as mediators of the virus-induced epithelial damage in RSV infected mice has been previously reported (3,4). Oxidative stress is one of the components of the pathophysiology of chronic obstructive pulmonary disease (5). RSV infection led to a ROS induction, which increases the expression of pro-inflammatory molecules such as interleukin-8 (IL-8), IL-6, CCL5 or CXCL10 (6). This production of pro-inflammatory cytokines induced by RSV infection resulted in type 1 and 2 cytokine imbalance. It was strongly suggested that excess type 2 and/or deficient type 1 immune responses were involved in the pathogenesis of RSV bronchiolitis (7).

Herbal medicines have been previously used in humans to treat medical illness or to improve physical performance. *Panax ginseng* is one of the most well-known herbal medicines that have been consumed for thousands of years. Experimental evidence suggests that ginseng modulates the host immune system and improves outcomes of inflammatory human diseases (8,9). Ginseng or its component ginsenoside protopanaxatriol was also reported to protect endothelial cells by scavenging hydroxyl radicals and modulating the antioxidant defense systems such as superoxide dismutase and glutathione peroxidase enzymes (10–12). Ginsenosides of ginseng were shown to protect human endothelial cells against influenza H9N2-induced inflammation and apoptosis (13). However, the potential antiviral effects of ginseng on RSV infection remain unknown.

In this study, we investigated the effect of Korean red ginseng extract (KRGE) on RSV replication, on RSV-induced cytokine expression, and RSV-induced cellular oxidative stress in a human epithelial cell line. In addition, we evaluated the possible *in vivo* antiviral effects of KRGE on clearing lung viral loads and host immune responses following RSV infection in a mouse model.

## Materials and methods

### Cells, virus and reagents

RSV A2 strain (a biosafety level 2 human pathogen) and HEp2 cells were used as previously described (14,15). The human alveolar type II-like epithelial cell line (A549 cell), was kindly provided by Dr Jae-Hyang Lim (Center for Inflammation, Immunity and Infection, Institute for Biomedical Sciences, Georgia State University). KRGE, a concentrated form of the commercial ginseng product was kindly provided by Korea Ginseng Corporation (Daejeon, Korea). Briefly, fresh roots of the *Panax ginseng* were washed, steamed at 100°C, and dried. The dried red ginseng roots were boiled in water for 3 h and the supernatants were concentrated. This preparation was designated as ‘KRGE’ and contained ~36% water content. Fetal bovine serum (FBS), penicillin-streptomycin, and Dulbecco’s modified Eagle’s medium (DMEM) were purchased from Gibco (Grand Island, NY, USA). 2′,7′-Dichlorodihydrofluorescein diacetate (H_2_DCFDA) was purchased from Molecular Probes (Carlsbad, CA, USA).

### Cell viability, RSV immunoplaque, and cytopathogenic effect (CPE) assays

Cell viability was determined using the 3-(4,5-dimethylthiazol-2-yl)-2,5-diphenyltetrazolium bromide (MTT) assay, which is based on the reduction of a tetrazolium salt by mitochondrial dehydrogenase in viable cells (16). Cell viability was expressed as a percentage of the control cells in the absence of RSV infection. RSV titers were determined by an immunoplaque assay (14,15). Virus stock or lung homogenates from infected mice were serially diluted and added to HEp2 cells that were grown to confluence. After 3 days of incubation, the infected cells were fixed with ice-cold acetone-methanol, and air-dried. Anti-F monoclonal antibody (Millipore, Billerica, MA, USA) and HRP-conjugated anti-mouse IgG antibodies (Southern Biotech, Birmingham, AL, USA) were used. Plaques were developed using a DAB substrate (Invitrogen, Grand Island, NY, USA). The CPE assay was performed as previously described (17). Treatment with KRGE was initiated 1 day prior to RSV infection. Confluent cell monolayer A549 cells grown in 96-well plates were infected with RSV, incubated for another two days in the presence or absence of KRGE, and virus-induced CPE was recorded.

### Reverse transcriptase-polymerase chain reaction (RT-PCR)

Total RNA was isolated using an RNeasy mini kit (Qiagen), according to the manufacturer’s instructions. Relative quantities of mRNA for each gene of interest were determined by semi-quantitative RT-PCR, as previously described (18). In brief, total RNA (1 μg) from each sample was used for the preparation of cDNA by using oligo(dT) primers and SuperScript III RT (Invitrogen). mRNA level of IL-6, IL-8 and 18S rRNA was determined by using the oligonucleotides as primers: IL-6 sense, 5′-GACAGCCACTCACCTCTTCA-3′ and antisense, 5′-CATCTTTGGAAGGTTCAGGTTGT-3′; IL-8 sense, 5′-CAGCCTTCCTGATTTCTGC-3′ and antisense, 5′-ACTTCTCCACAACCCTCTGC-3′; 18S rRNA sense, 5′-ATCCTGCCAGTAGCATATGC-3′ and antisense, 5′-ACCCGGGTTGGTTTTGATCTG-3′. RT-PCR products were visualized on 2% agarose gels containing ethidium bromide. 18S rRNA was the internal control. The relative quantity of PCR products was expressed as fold increase relative to phosphate-buffered saline (PBS) controls.

### Intracellular ROS measurement and image analysis

The change in fluorescence resulting from the oxidation of the fluorescent probe H_2_DCFDA was used to evaluate the level of intracellular ROS (19). After RSV and KRGE treatments, the cells were incubated with 50 mM of the fluorescent probe H_2_DCFDA. The degree of fluorescence was detected at an excitation and emission of 485 and 535 nm, respectively, using a microplate spectrofluorometer (Molecular Devices Corp., Sunnyvale, CA, USA). Coverslip-loaded 6-well plates were used for image collection during cell culture. After various treatments, H_2_DCFDA solution was added to each well of the plate, which was incubated for 2 h at 37°C. Images of the stained cells were collected using a fluorescence microscope (Nikon, Melville, NY, USA).

### Treatment of mice with KRGE and RSV virus

KRGE was dissolved in sterile PBS and filtered through 0.4 μm Millipore membrane. For animal experiments, 8- to 10-week-old female BALB/c mice (Harlan Laboratories, Indianapolis, IN, USA) were lightly anesthetized by isoflurane and then orally administered KRGE in water feeding at a dose of 25 mg/kg/day for 60 days. In addition, 8- to 10-week-old female mice serving as controls were treated with PBS at the beginning of this experiment. This dose was based on previous studies with regard to oral intake of ginseng extracts in humans and mouse/kg of body weight (20,21). To determine the effects of KRGE treatment on RSV infection, the mice (n=5/group) were anesthetized by isoflurane inhalation and intranasally infected with RSV A2 (2×10^6^ PFU) in a volume of 50 μl. All the animal experiments presented in this study were approved by the Georgia State University (GSU) Institutional Animal Care and Use Committee review board and carried out in the animal biosafety level 2 facility.

### Lung virus titer and cytokine assays

Mice were anesthetized with isoflurane and exsanguinated after severing of the right caudal artery. The individual lungs were removed aseptically at day 5 post challenge, and lung extracts were prepared as homogenates after challenge using frosted glass slides (15). Lung homogenates for viral titers from individual mice were prepared using a mechanical tissue grinder with 1.5 ml of PBS. The homogenates were centrifuged at 1,100 × g for 10 min to collect supernatants. Virus titers in the supernatants were determined by immunoplaque assay. Ready-Set-Go IL-4, IL-5, IL-13, tumor necrosis factor-α (TNF-α), and interferon-γ (IFN-γ) kits (eBioscience) were used to detect cytokine levels in bronchoalveolar fluids (BALF) following the manufacturer’s recommended procedures (22).

### Preparation of bronchoalveolar lavage (BAL) and flow cytometric analysis

Five days after RSV infection, the mice were sacrificed using carbon dioxide inhalation to collect BAL fluids and lung samples. BAL fluid samples were obtained by infusing 1 ml of PBS into the lungs via the trachea using a 25-gauge catheter (Exelint International Co., Los Angeles, CA, USA) as previously described (23,24). The cells from BAL fluids were pooled (n=5) and then stimulated with phorbol myristate acetate (50 ng/ml) and ionomycin (500 ng/ml) for 4 h. After staining with surface antibodies (anti-CD3, CD4, CD8α, CD11b and CD11c antibodies from eBiosciences), intracellular IFN-γ cytokine staining was followed according to the manufacturer’s instructions (BD Cytofix/Cytoperm™ Fixation/Permeabilization Solution kit). The percentage of gated cells was calculated by FlowJo software (Tree Star Inc., Ashland, OR, USA).

### Statistical analysis

Data are expressed as the means ± standard error (SE), and the results were taken from at least three independent experiments performed in triplicate. The data were analyzed by the Student’s t-test to evaluate significant differences. P<0.05 was considered statistically significant.

## Results

### Influence of KRGE on RSV replication in human epithelial cells

**Figure 1:**
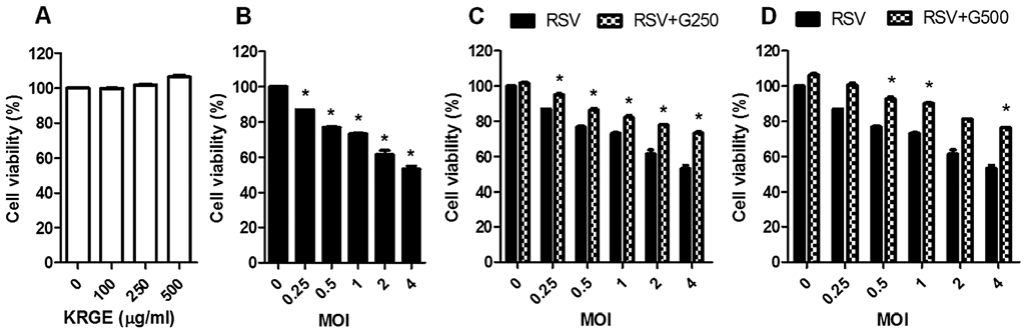
Influence of Korean red ginseng extract (KRGE) on respiratory syncytial virus (RSV)-induced cytopathogenic effects. (A) Influence of KRGE concentrations on the growth of A549 cells. KRGE did not affect A549 cells. Values are the means ± standard error (SE). (B) Effects of different RSV multiplicities of infection (MOIs) on the viability of A549 cells. Values are the means ± SE. ^*^P<0.05 vs. mock control. (C and D) Influence of different KRGE concentrations on the viability of A549 cells infected with RSV A2 virus at different MOIs 48 h post-infection. A549 cells were continuously treated with KRGE starting 1 day prior to infection and during the infection period. Cell survival was increased at concentrations of 250 and 500 μg/ml. Values are the means ± SE. ^*^P<0.05 vs. RSV-infected control. G250, 250 μg KRGE/ml; G500, 500 μg KRGE/ml.

**Figure 2:**
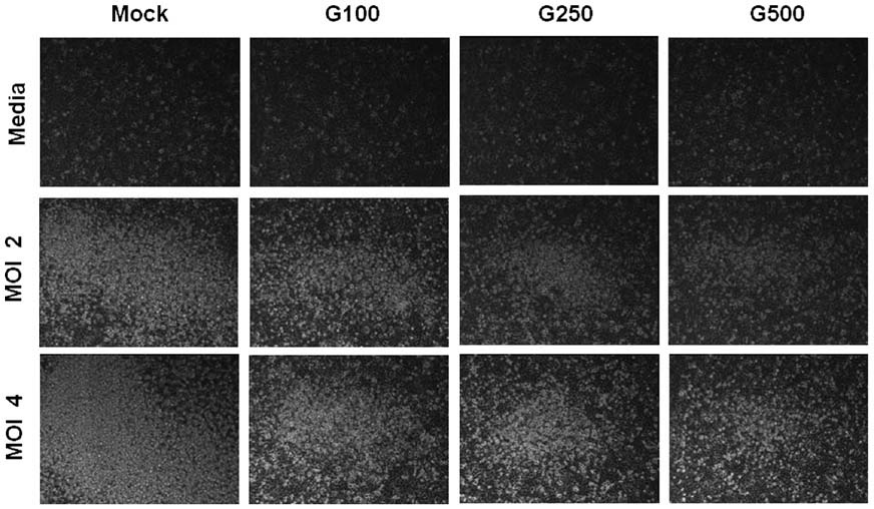
Microscopic observation of Korean red ginseng extract (KRGE) effects on respiratory syncytial virus (RSV)-induced cell death. A549 cells were mock or RSV-infected at different multiplicities of infection (MOIs). White spots are dead floating cell aggregates observed under the microscope. Mock, G100, G250 and G500 indicate the contraction of KRGE 0, 100, 250 and 500 μg/ml. MOI 2 or 4 indicate the amount of RSV used to infect A549 cells.

**Figure 3:**
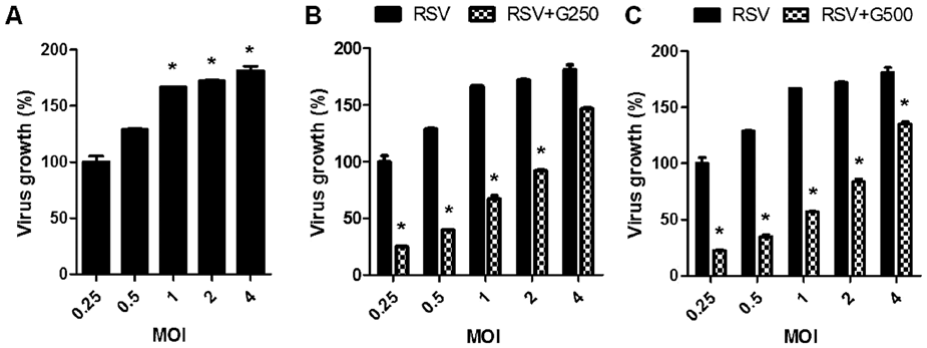
Korean red ginseng extract (KRGE) shows antiviral activity on respiratory syncytial virus (RSV) in human epithelial cells. (A) Growth of RSV in A549 human epithelial cells. Values are the means ± standard error (SE). ^*^P<0.05 vs. RSV-infected control at a multiplicity of infection (MOI) of 0.25. (B and C) Influence of different KRGE concentrations on the growth of RSV in A549 cells infected with RSV at different MOIs 48 h post-infection. Values are presented as means ± SE of three independent experiments and are expressed as the percentage of virus growth relative to the value of RSV infection at a MOI of 0.25. ^*^P<0.05 vs. RSV-infected control.

To investigate the effects of KRGE on RSV replication in human epithelial cells, confluent A549 cells were infected with RSV at different multiplicity of infection (MOI) in the presence or absence of KRGE treatment. KRGE did not affect A549 cell viability (Fig. 1A). After RSV infection, A549 cells exhibited marked morphological changes, suggesting cell death in a MOI-dependent manner (Fig. 1B), such as cell rounding and detachment (Fig. 2). When A549 cells were treated with KRGE, RSV-induced cell death was partially reduced and cell survival was increased at the concentrations of 250 and 500 μg/ml (Fig. 1C and D). CPE was also examined under the microscope after KRGE treatment and RSV infection. KRGE treatments resulted in lower CPE levels as evidenced by reduction of the number of floating cells due to RSV infection (Fig. 2). Higher viral growth was observed by increasing the MOI (Fig. 3A). In the presence of KRGE, much lower levels of RSV growth were observed. The magnitude of growth inhibition was more pronounced at lower MOIs of RSV (Fig. 3B and C). Therefore, these results showed that KRGE treatment reduced cell death by RSV infection. More importantly, KRGE treatment significantly inhibited the growth of RSV *in vitro* A549 cell cultures.

### Influence of KRGE on inflammatory cytokine production in human epithelial cells

**Figure 4:**
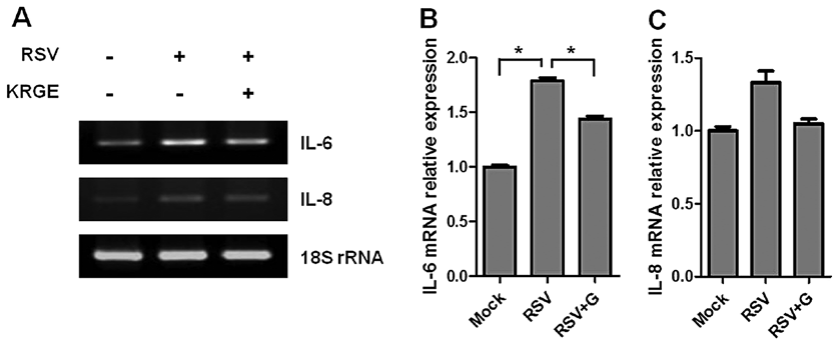
Korean red ginseng extract (KRGE) inhibits respiratory syncytial virus (RSV)-induced cytokine production. A549 cells were mock-infected or infected with RSV. A549 cells were treated with KRGE 1 day prior to infection and during the infection period of 2 days. Cytokine mRNA levels were determined by semi-quantitative RT-PCR. (A) RT-PCR cDNA bands on the agarose gel. (B) Interleukin-6 (IL-6). (C) IL-8. Mock, media only; RSV, RSV infection; RSV + G, RSV and KRGE (500 μg/ml). Values are the means ± standard error (SE). ^*^P<0.05.

To investigate RSV-induced cytokine expression, confluent cell layers were infected with RSV at a MOI of 1 in the presence or absence of KRGE treatment. Total RNA was extracted from the cells, and the gene expression was evaluated by RT-PCR. RSV-infected A549 cells significantly increased the expression of IL-6 and IL-8 cytokines compared to the mock-treated cells. IL-6 was expressed more prominently than IL-8 cytokine. KRGE treatment resulted in lower levels of RSV-induced expression of IL-6 cytokine gene in RSV-infected human epithelial cells (Fig. 4). KRGE treatment also showed a trend of lowering IL-8 cytokine. Therefore, these results suggested that KRGE inhibits the production of inflammatory cytokines induced by RSV infection.

### Influence of KRGE on ROS formation in human epithelial cells

**Figure 5:**
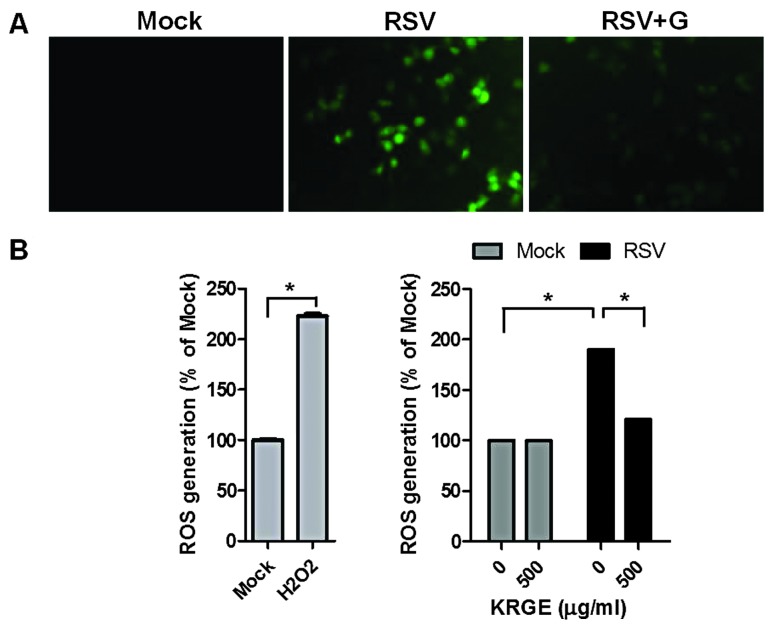
Korean red ginseng extract (KRGE) inhibits respiratory syncytial virus (RSV)-induced ROS formation in A549 cells. A549 cells were mock-infected or infected with RSV in the presence or absence of KRGE. (A) Fluorescence microscopic image of RSV-induced ROS formation. (B) RSV-induced ROS generation and effects of KRGE on ROS formation as determined by staining with fluorescent dye, dichlorodihydrofluorescein diacetate. The degree of fluorescence was detected at a 485 nm excitation and a 535 nm emission using a microplate spectrofluorometer. Mock, media only; RSV, RSV infection; RSV + G, RSV and KRGE (500 μg/ml). Values are the means ± standard error (SE). ^*^P<0.05.

Oxidative stress-mediated events are known to be involved in virus infection mechanisms and correlated with the release of inflammatory mediators from epithelial cells (25). Intracellular ROS levels in RSV-infected A549 cells were determined using the ROS-sensitive fluorescent probe H_2_DCFDA. KRGE treatment did not affect ROS formation of the non-infected cells. The fluorescence intensity of the dichlorofluorescein diacetate stain was significantly enhanced in RSV-infected human epithelial cells. Of nore, KRGE treatment significantly inhibited RSV-induced ROS formation as indicated by lowering green fluorescence intensity (Fig. 5). These results suggested that ginseng treatment inhibits RSV-induced ROS generation.

### KRGE protects mice from RSV infection

**Figure 6:**
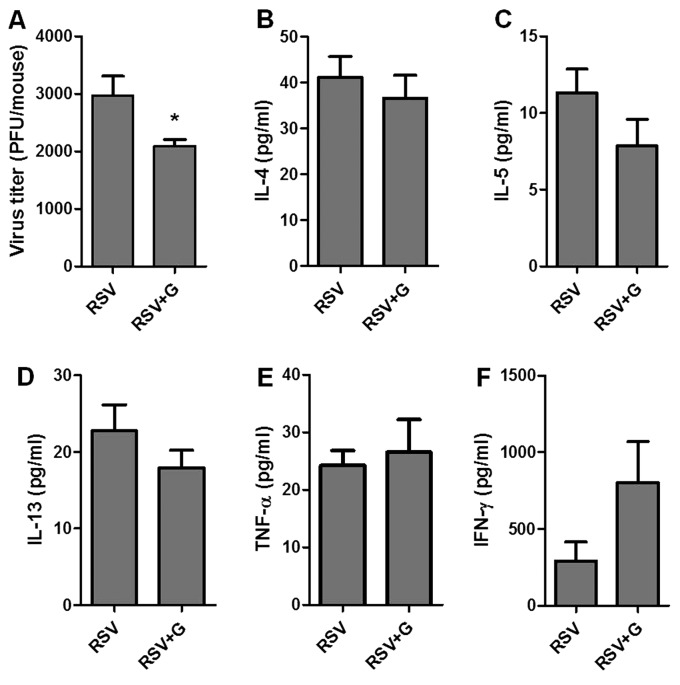
*In vivo* effects of Korean red ginseng extract (KRGE) on lung viral titers and cytokines in mice infected with respiratory syncytial virus (RSV). (A) Lung viral titers and (B) bronchoalveolar fluid (BALF) cytokine levels of interleukin-4 (IL-4), (C) IL-5, (D) IL-13, (E) tumor necrosis factor-α (TNF-α) and (F) interferon-γ (IFN-γ) were determined at day 5 post-RSV infection (n=5). RSV, naïve mice infected with RSV; RSV + G, KRGE was administered orally at a dose of 25 mg/kg/day for 60 days and then mice were infected with RSV. Values are the means ± standard error (SE). ^*^P<0.05 vs. RSV-infected control.

**Figure 7:**
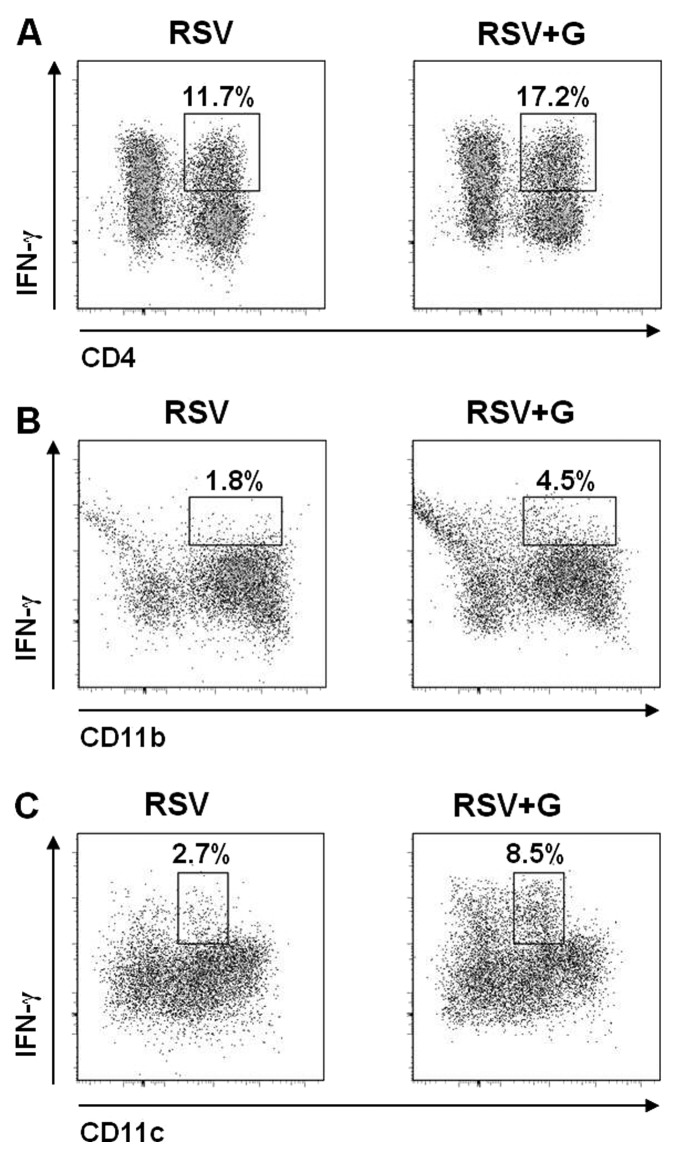
*In vivo* effects of Korean red ginseng extract (KRGE) on phenotypes of bronchoalveolar cells producing interferon-γ (IFN-γ). KRGE was administered orally at a dose of 25 mg/kg/day for 60 days and then mice were challenged with respiratory syncytial virus (RSV) (2×10^6^ PFU). Bronchoalveolar lavage (BAL) was collected 5 days after RSV challenge, and BAL cells were pooled (n=5 BALB/c mice/group), and stained with CD3, CD4, CD8, CD11b, CD11c and IFN-γ antibodies, and analyzed by flow cytometry. The cells were first gated by their cell size and IFN-γ-producing (A) CD3^+^CD4^+^ T cells, (B) CD11b^+^ cells and (C) CD11c^+^ cells were observed. The numbers in dot plots indicate the cell percentages of the double-positive population. Representative data are shown of two independent sets of experiments (two sets of experiments n=10). RSV, naïve mice challenged with RSV; RSV + G, KRGE was administered orally at a dose of 25 mg/kg/day for 60 days and then mice were challenged with RSV.

To better understand the potential role of KRGE in conferring protection against RSV infection, we tested the *in vivo* effects of KRGE on viral infection in a mouse model. BALB/c mice were orally administered 25 mg/kg/day of drinking water for 60 days. KRGE and mock-treated mice were infected by intranasal inoculation of RSV (2×10^6^ PFU/mouse). Lungs and BALF were collected at day 5 post-infection for analysis of lung viral titers and cytokine levels. The group of mice that were orally treated with ginseng for 60 days showed a lower level of lung viral titers compared to that in the RSV-infected naïve control (Fig. 6A). In addition, the levels of pro-inflammatory cytokines IL-4, IL-5 and IL-13 were found to be lower in the KRGE-treated group following RSV infection compared to the untreated control, with no statistical significance being observed (Fig. 6). Levels of TNF-α in BALF were similar in the two groups. The levels of antiviral cytokine IFN-γ showed a higher level in the KRGE-administered mice compared to the infected naïve control mice (Fig. 7), with no statistical significance being observed.

### KRGE increases antiviral IFN-γ-producing cells in mice following RSV infection

**Figure 8:**
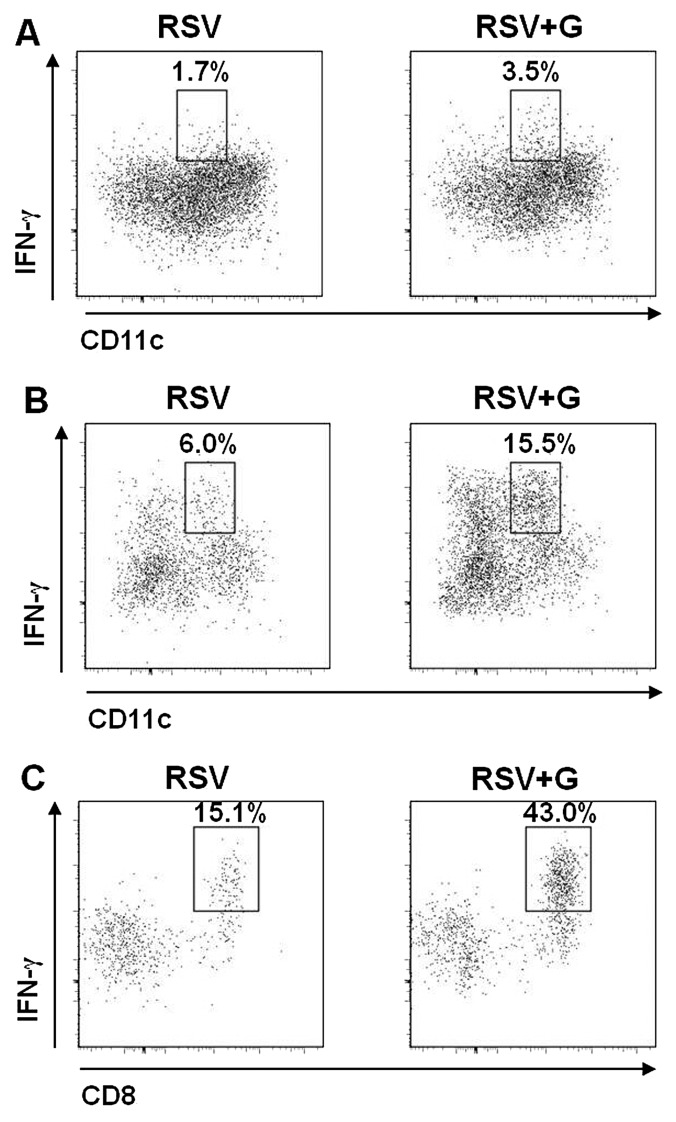
Phenotypes of CD11c^+^ dendritic cells producing interferon-γ (IFN-γ) in bronchoalveolar lavage (BAL) fluids from mice with Korean red ginseng extract (KRGE) treatment, followed by respiratory syncytial virus (RSV) infection. BAL cells were harvested, pooled (n=5/BALB/c mice group), and stained as described in Fig. 7. IFN-γ producing CD11c^+^ cells were analyzed. (A) CD11c^+^IFN-γ^+^ cells in CD11b^+^ granulocytes. (B) CD11c^+^IFN-γ^+^ cells in CD11b^−^ granulocytes. (C) CD8^+^IFN-γ^+^ cells in CD11b^−^CD11c^+^ granulocytes. The numbers in the dot plots indicate the cell percentages of double positive population. The numbers in dot plots indicate the cell percentages of the double-positive population. Representative data are shown of two independent sets of experiments (two sets of experiments n=10). RSV, naïve mice challenged with RSV; RSV + G, KRGE was administered orally at a dose of 25 mg/kg/day for 60 days and then mice were challenged with RSV.

To better understand the potential role of KRGE in conferring protection against RSV infection, the levels of IFN-γ-producing cells in BALF were examined by intracellular IFN-γ cytokine staining at day 5 post infection with RSV. From the flow cytometric analysis of pooled BAL cells of 5 mice in each group, we observed that oral administration of KRGE to mice showed higher levels of various phenotypic cells producing IFN-γ antiviral cytokine following RSV infection (Figs. 7 and 8). A higher level of IFN-γ-producing CD4^+^ T cells was detected in BALF from mice that were orally treated with KRGE compared to that from the control group without KRGE following RSV infection (11.7 vs. 17.2%) (Fig. 7A). Additionally, CD11b^+^ granulocytes producing IFN-γ were higher in the KRGE-treated mouse group than those in the untreated mice with RSV infection (1.8 vs. 4.5%) (Fig. 7B). In particular, CD11c^+^ dendritic cells producing IFN-γ were found to be 3-fold higher in BALF samples of KRGE-treated mice compared to that from the control group following RSV infection (2.7 vs. 8.5%) (Fig. 7C). Sub-populations producing IFN-γ were further dissected with additional phenotypic markers CD11b and CD8α (Fig. 8). CD11c^+^CD11b^+^IFN-γ^+^ granulocytes were 2-fold higher in the KRGE-treated group compared to the non-treated controls, although these granulocytes are not a major population producing IFN-γ (Fig. 8A). The majority of granulocytes expressing IFN-γ in the KRGE-treated group were found to be CD11b^−^CD11c^+^IFN-γ^+^ dendritic cells (Fig. 8B), expressing CD8α^+^ (Fig. 8C). The lymphocytic marker CD8α^+^ expressing CD11c-positive dendritic cells is known to contribute to inducing T-helper type 1 (Th1) immune responses (26). Thus, KRGE treatment may stimulate innate and adaptive Th1-type immune responses by stimulating CD11b^−^CD8α^+^CD11c^+^ dendritic cells.

## Discussion

Ginseng has been known to have various immunomodulatory functions. However, the potential anti-viral activity, oxidative stress, inflammatory cytokine production, and immunomodulatory effects of *Panax ginseng* on RSV infection remain unknown. In the present study, we showed that KRGE inhibited the replication of RSV, RSV-induced cell death, expression of pro-inflammatory cytokines, and suppressed RSV-induced ROS formation in human epithelial cells. Oral intake of ginseng products is the most common means of consumption as a nutrient supplement in healthy individuals. Therefore, we assessed the potential antiviral effects and immunomodulatory functions of KRGE following RSV viral infection in a mouse model. Oral administration of KRGE to mice conferred moderate but significant resistance to RSV infection. Therefore, the present study provides evidence that KRGE has antiviral activity against RSV *in vitro* and *in vivo*. Anti-RSV activity of KRGE has a high significance considering the fact that RSV is the most frequent viral cause of respiratory diseases, asthma, and chronic obstructive pulmonary disease exacerbations early and later in life (27).

The results showed that RSV caused significant cell death following RSV infection in a dose-dependent manner, presumably due to RSV-induced oxidative damage to the cells (3). Cellular oxidative damage due to RSV infection was reported as a result of inducing an imbalance between ROS production and antioxidant cell defenses (3). Various cytokines and chemokines are known to be expressed during RSV infection, playing a central role in pathogenesis (28). These effects have been associated with activation of the transcription factor nuclear factor-κB and mitogen-activated protein kinase through oxidant-dependent mechanisms (25). KRGE treatment partially protected the cell death from RSV infection. The underlying mechanisms by which KRGE treatment improves cell survival during RSV infection remain to be defined. KRGE treatment significantly inhibited the *in vitro* growth of RSV by 3–5-fold as well as the production of RSV-induced pro-inflammatory cytokines and ROS formation. A possible mechanism is that the KRGE-mediated inhibition of RSV replication in alveolar epithelial cells may have resulted in a reduction of ROS and pro-inflammatory cytokine production, which subsequently resulted in improving cell survival. Alternatively, an anti-oxidant effect by KRGE may have independently contributed to improving cell survival protection. Thus, it is hypothesized that KRGE may exhibit antiviral activity against RSV in various ways during RSV infection.

Findings of a previous study demonstrated that oral administration of KRGE prior to infection significantly increased survival rates of mice following infection with 2009 pandemic H1N1 virus (20). Results of the present study have shown that oral administration of KRGE to mice prior to infection resulted in lowering lung viral loads subsequent to RSV infection. This reduction is highly significant as oral intake of KRGE protects the local infection of RSV in lungs. The anti-inflammatory effects of KRGE on inflammatory cytokines in BALF samples were minimal, likely because RSV is not highly pathogenic to mice although RSV replicates to a certain level (29).

The ginsenosides, the constituents of KRGE were shown to reduce IL-4 production but increase IFN-γ production, resulting in Th1-type immune responses in an ovalbumin-induced murine model of asthma (30). Dendritic cells are crucial in determining the fate of naïve T cells, whether Th1 or Th2 cells (26). However, the effects of ginseng on dendritic cells and resulting T-cell responses remain to be determined. A significant finding in this study on RSV is that KRGE oral treatment increased IFN-γ production in BALF following RSV infection. A detailed analysis of BALF cells provided evidence that KRGE may stimulate the production of IFN-γ-producing CD4 T lymphocytes as well as granulocytes such as CD11b^+^ and CD11c^+^ cells. Particularly, the increase in CD11b^−/low^CD11c^+^CD8α^+^ cell populations was prominent as a result of KRGE treatment, which may contribute to the production of IFN-γ and the increased induction of CD4 T cells producing IFN-γ. The cellular phenotypes of IFN-γ production and RSV pathogenesis as a result of ginseng treatment may be different from those by RSV-infected epithelial cells that were associated with severe pneumonia due to RSV infection (31). Taken together, this study suggests a mechanism that ginseng may have antiviral activity against respiratory virus by modulating host cellular phenotypes producing cytokines.

In conclusion, the modulation of oxidative stress is a potential novel pharmacologic approach that may be used to ameliorate RSV-induced acute lung inflammation. Ginseng inhibited RSV-induced cellular oxidative damage and blocked the induction of RSV-induced pro-inflammatory gene expression in the human alveolar epithelial cell line. Furthermore, ginseng suppressed lung viral titer and contributed to protective immunity by enhancing IFN-γ production following RSV viral infection in an experimental RSV infection murine model. Based on the results, although the exact underlying anti-virus mechanism of ginseng remains to be determined, consumption of ginseng in healthy individuals would have beneficial effects in the prevention of unexpected RSV infections and/or reduction of the severity of RSV disease.
